# Stemming Epigenetics in Marine Stramenopiles

**DOI:** 10.2174/138920211796429727

**Published:** 2011-08

**Authors:** Florian Maumus, Pablo Rabinowicz, Chris Bowler, Maximo Rivarola

**Affiliations:** 1 Unité de Recherche en Génomique-Info, UR 1164, INRA Centre de Versailles-Grignon, Versailles, France; 2 Institut de Biologie de l'Ecole Normale Supérieure, Section de Génomique Environnementale et Evolutive, CNRS UMR 8197 INSERM U1021, Paris, France; 3 Institute for Genome Sciences, Department of Biochemistry and Molecular Biology, University of Maryland, School of Medicine, BioPark Building II, 801 West Baltimore Street, Baltimore, MD 21201, USA; 4 Instituto Nacional de Tecnología Agropecuaria (INTA), Instituto de Biotecnología (CNIA), CC 25, Castelar (B1712WAA), Buenos Aires, Argentina

**Keywords:** Marine stramenopiles, epigenomics, DNA methylation, chromatin, diatom, genomics, Small RNA, Brown algae, Transposable elements.

## Abstract

Epigenetics include DNA methylation, the modification of histone tails that affect chromatin states, and small RNAs that are involved in the setting and maintenance of chromatin modifications. Marine stramenopiles (MAS), which are a diverse assemblage of algae that acquired photosynthesis from secondary endosymbiosis, include single-celled organisms such as diatoms as well as multicellular forms such as brown algae. The recent publication of two diatom genomes that diverged ~90 million years ago (mya), as well as the one of a brown algae that diverged from diatoms ~250 Mya, provide a great system of related, yet diverged set of organisms to compare epigenetic marks and their relationships. For example, putative DNA methyltransferase homologues were found in diatoms while none could be identified in the brown algal genome. On the other hand, no canonical DICER-like protein was found in diatoms in contrast to what is observed in brown algae. A key interest relies in understanding the adaptive nature of epigenetics and its inheritability. In contrast to yeast that lack DNA methylation, homogeneous cultures of diatoms constitute an attractive system to study epigenetic changes in response to environmental conditions such as nutrient-rich to nutrient-poor transitions which is especially relevant because of their ecological importance. *P. tricornutum* is also of outstanding interest because it is observed as three different morphotypes and thus constitutes a simple and promising model for the study of the epigenetic phenomena that accompany cellular differentiation. In this review we focus on the insights obtained from MAS comparative genomics and epigenomic analyses.

## INTRODUCTION

Epigenetics has different biological meanings for different people. It was historically defined by Conrad H. Waddington as how the “canalization” of genotypes can give rise to inheritable phenotypes through “genetic assimilation”. This concept, which involves evolutionary feedback through natural selection of acquired traits was therefore reintroducing the Lamarckian concept into adaptive evolution. Decades later, Arthur Riggs and collaborators defined epigenetics as “the study of mitotically and/or meiotically heritable changes in gene function that cannot be explained by changes in DNA sequence”. Rather than pointing to one defined biological phenomena, the contemporary use of the word “epigenetics” embraces different basic biological mechanisms that were found to participate in setting and maintaining heritable changes that do not rely on changes in DNA sequence. These encompass various phenomena that modify chromatin including the modification of histone marks, DNA methylation, RNA intereference (RNAi), and changes in higher-order structure of the chromosomes in the nucleus. These processes play key roles in generating and maintaining specific chromatin states that impact its compaction and accessibility and thus affect the readout of the underlying DNA sequence. In some cases, such modifications can be transmitted to subsequent generations. For instance, it was found that the phenotype of a peloric reversible mutant of *Linaria vulgaris* was due to extensive DNA methylation and transcriptional silencing at the *Lcyc* gene, which controls floral dorsoventral asymmetry [1]. Such inherited epigenetic marks are often referred to as “epimutations”. By contrast, some chromatin marks can be highly dynamic. For example, the phosphorylation of the variant histone H2AX after double-strand break is only transient [2]. As a consequence, an incorrect but widespread use of the word epigenetics refers to the study of these chromatin-related processes, whether their outcomes are inherited or not. Therefore, the temporary chromatin modifications accompanying processes such as DNA repair, DNA replication, alternative splicing [3], and reprogramming [4] are also sometimes dubbed “epigenetic” phenomena [5]. One reason for this is that in many organisms, including those that are the subject of this review, there are very few studies of the heritability of such phenomena.

In eukaryotes, cytosine methylation is a common epigenetic mark that can impact gene expression. It is essential for various biological processes, including transposon silencing [6], imprinting [7], and X chromosome inactivation [8, 9]. The addition of a methyl group on cytosine occurs specifically in the symmetric CG and CHG contexts (in which H = A, T or C), and the asymmetric CHH context [10, 11]. “*De novo*” methylation acts on un-methylated residues in a small RNA-guided fashion known as RNA-dependent DNA methylation (RdDm) [12, 13]. RdDm was first discovered in plants and acts through small double-stranded RNA (dsRNA) that guide DNA methylation at specific loci and in most cases represses gene expression [14]. *De novo* DNA methylation also occurs through spreading from existing methylated loci [15]. Upon DNA replication, methylated symmetric DNA sites become hemi-methylated and are recognized by the maintenance DNA methylation machinery that methylates the corresponding cytosine in the newly synthesized strand [16].

Histone tails can undergo various post-translational modifications such as methylation, acetylation, phosphorylation, and ubiquitylation. Each histone (H2A, H2B, H3, and H4) and histone variant in a nucleosome can carry different modifications. Additional layers of complexity come for example from the fact that a histone can be modified on various residues and that different lysines can be mono-, di-, or trimethylated. As a result, over sixty different modified histones have been reported from different organisms [17], leading to a highly variable pool of modified nucleosomes. Each type of nucleosome can be organized into clusters along the chromosomes and index chromatin with reference to the associated DNA sequence to transmit specific information [18], positively or negatively impacting the activity of the transcriptional machinery either by direct interaction and/or by triggering chromatin remodeling. This vast array of modifications also provides an enormous potential for tuning transcriptional levels in response to signaling conditions within a cell or in different cell types. Through the S phase, at the replication fork, the parental nucleosomes are disrupted into two H2A–H2B dimers and an (H3–H4)2 tetramer or two H3–H4 dimers. Their transfer or recycling onto the newly synthesized daughter strands provides a first source of histones and the nucleosomal density is restored by assembly of new histones on the duplicated material. Parental histone post translational modifications potentially preserved during transfer can be used as a template to reproduce marks on newly incorporated histones [19].

The RNAi machinery in eukaryotes is involved in transcriptional and translational regulation as well as in the primary defense against viruses and transposable elements. This regulatory system relies on the production and action of various classes of small (~20-30 nucleotides) RNA with specific functions. In addition to guiding DNA methylation, other classes of small RNAs including micro RNAs (miRNA) are involved in post-transcriptional gene regulation by directing the cleavage or translational inhibition of transcripts through complete or partial complementarity of their sequence to the target sequence [20, 21].

These processes are the subject of intense studies in model organisms including plants, fungi, and animals where they were found to accomplish key functions. The availability of complete genome sequences and new technologies such as whole-genome tiling arrays and new generation sequencing (NGS) have recently bolstered genome-wide analysis of epigenetic marks by enabling the realization of protocols such as ChIP on chip (a technique which combines chromatin immunoprecipitation to isolate DNA associated with specific proteins such as modified histones, and its quantification using microarray technology), ChIP-seq (similar to ChIP on chip but with the use of new generation sequencing to quantify DNA), RNA-seq (also called "Whole Transcriptome Shotgun Sequencing”, uses new sequencing technologies to quantify a library of cDNA), small RNA-seq (similar to RNA-seq but starts with a library of only small RNAs), BS-seq (also referred to as “Whole genome bisulfite sequencing”, is a high-throughput genome-wide analysis of DNA methylation), and so on [22-24]. Although huge efforts were recently contributed to deciphering the DNA methylome of various species [11, 10, 25, 26] at single-base resolution, little is known about the occurrence and functions of epigenetic regulation in eukaryotes beyond the animal, plant and fungal lineages.

**Fig. (1):**
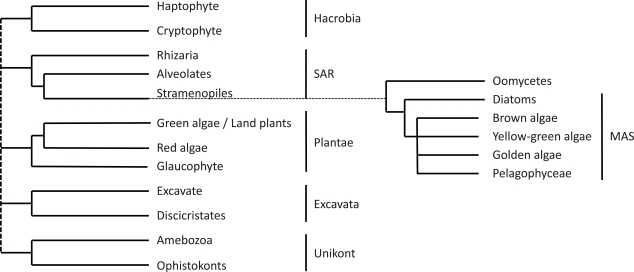
**Stramenopiles are a major lineage of eukaryotes.** Consensus cladogram of selected eukaryotes highlighting five major eukaryotic supergroups with support in recent phylogenetic studies [28, 31, 32]. The dotted polytomy indicates uncertainty regarding the order of early branching events. The inset is a consensus cladogram of selected stramenopiles.

Stramenopiles (also called heterokonts) are a major line of eukaryotes that comprises mostly autotrophic algae such as diatoms and brown algae, but also heterotrophs such as oomycetes, including plant parasites such as *Phytophthora* (Fig. **1**). They derive from a secondary endosymbiotic event in which an extant heterotrophic eukaryote either engulfed or was invaded by a red alga [27]. As of 2011, stramenopiles are supposed to belong to a monophyletic eukaryotic supergroup dubbed SAR after the major groups that constitute it: Stramenopiles, Alveolates, and Rhizaria [28] (Fig. **1**). It has also been postulated, as the ‘Chromalveolata’ supergroup hypothesis [29], that a single secondary endosymbiosis event, about 1,300 Mya [30], originated the presence of red algal plastids in members of both the stramenopiles and the alveolates and in more distantly related groups such as haptophytes and cryptophytes but not in Rhizaria. However, the analysis of recent sequencing data suggests that chromalveolates constitute a paraphyletic group [31-33]. The origins, types, and acquisition times of secondary plastids in diverse eukaryotic groups including stramenopiles are still the subject of open debate [33-35].

**Fig. (2):**
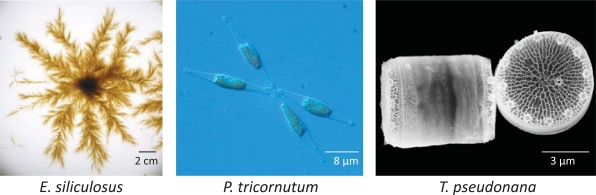
**Sequenced MAS species.** Images from *Ectocarpus siliculosus* (Delphine Scornet, CNRS, Roscoff, France), *Phaeodactylum tricornutum* (Alessandra De Martino, CNRS, Paris, France), and *Thalassiosira pseudonana* (Nils Kröger, Georgia Institute of Technology, Atlanta, USA).

Diatoms (*Bacillariophyceae*) are among the most successful and diversified groups of photosynthetic eukaryotes, with possibly over 100,000 extant species [36]. The contribution of diatom photosynthesis to marine primary productivity has been estimated to be around 40% [37-40]. They are traditionally divided into two orders: the centric diatoms which are radially symmetrical and are thought to have arisen around 180 Million years ago (Mya), followed by the pennate diatoms around 90 Mya which are bilaterally symmetrical [41]. A less ubiquitous group, the brown algae (*Phaeophyceae*) such as giant kelps, are mostly restricted to near-shore lines and rocky areas although some genus such as *Sargassum* are also found free-floating. Brown algae are an essential source of ecological habitats and food for zooplankton, crustaceans, and fish. Interestingly, brown algae represent one of the few eukaryotic lineages that evolved multicellularity. Due to their evolutionary, biological and ecological interest, the genome sequences of three marine stramenopiles (MAS) (two highly divergent diatoms and a brown algae) have recently been published. *Thalassiosira pseudonana* (32 Mb) was the first diatom and eukaryotic marine phytoplankton genome to be sequenced [42] (Fig. **2**; Table **1**). *T. pseudonana* is a centric diatom present throughout the world’s oceans and for which sexual reproduction has never been reported. The second diatom genome sequenced belongs to the pennate diatom *Phaeodactylum tricornutum* (27 Mb) [43]. *P. tricornutum* is mostly confined to coastal habitats and also appears to lack  a sexual life cycle (Fig. **2**; Table **1**). Both genomes are relatively small respect to other diatoms, which can reach up to 50 Gb in *Coscinodiscus sp*. [44]. The brown algal genome is from the filamentous seaweed *Ectocarpus siliculosus* (214 Mb) [45], which is now considered as a model for the brown algae (Fig. **2**; Table **1**). Its genome sequence provides new opportunities for the application of genomic and epigenomic approaches to study the brown algal biology. More recently, a sequence-tagged genetic map substantially improved the assembly of the *E. siliculosus* genome sequence [46]. These three genomes constitute levers enabling comprehensive studies of epigenetic regulation in stramenopiles.

In this review, we describe the tools available for genetic and epigenetic studies of MAS, highlight some of the most important findings coming from genomic and comparative genomic analyses in these species, and provide an overview and perspectives on our current knowledge about epigenetic mechanisms in sequenced MAS species.

## THE MAS TOOLBOX

On the route towards turning MAS species into model systems, the research community has developed various means to carry out functional genomic studies in MAS organisms with sequenced genomes. Extensive cDNA sequence databases containing over 200,000 *P. tricornutum* and *T. pseudonana* expressed sequence tags (ESTs) from cells grown under 16 and 7 different conditions, respectively [47] (http://www.diatomics.biologie.ens.fr/EST3/) constitute an important resource for gene expression analysis. Many of those culture conditions reflect ecologically significant stresses that commonly occur in contemporary oceans, such as nitrate and iron limitations [48]. In addition, over 90,000 *E. siliculosus* ESTs have been obtained from 4 different life stages and one stress condition [49]. EST data from *E. siliculosus* were used to design a microarray containing features representing some 17,000 genes, and which has been used to measure gene expression levels during acclimation to three different abiotic stress conditions (hyposaline, hypersaline, and oxidative stress) [49] (http://ww.sb-roscoff.fr//UMR7139/ectocarpus/transcriptomics/). Whole genome tiling microarrays have also been designed for *T. pseudonana* [50], *P. tricornutum* (Maumus *et al*., unpublished) and *E. siliculosus* [45].

Functional analyses in diatoms are further bolstered by a molecular toolbox providing protocols and materials for stable transformation of the nuclear genome by biolistics [51, 52], gene cloning using diatom-optimized gateway vectors for protein subcellular localization and immunodetection [53], and targeted gene silencing strategies based on RNA interference mechanisms [54]. Classical genetic techniques, genetic markers and mutagenesis protocols are also available for *E. siliculosus* [55].

## INSIGHTS FROM MAS GENOMICS

Comparative analyses of diatom genomes revealed that *P. tricornutum *has fewer genes than *T. pseudonana *(10,402 opposed to 11,776) and no major synteny could be detected between the two genomes [43]. *T. pseudonana *genes show an average of ~1.52 introns per gene as opposed to 0.79 in *P. tricornutum*, suggesting recent widespread intron gain in the centric diatom [56]. Of greater significance, diatom genomes have provided a wealth of information about the processes of gene transfer (EGT) from endosymbiotic organelles to the host nuclear genome that occurred during stramenopile evolution. For example, the *T. pseudonana* genome encodes six proteins, which are most closely related to genes from the nucleomorph (remnant nucleus of algal endosymbiont [57]) genome of the cryptophyte* Guillardia theta*. Four of these genes were also found in red algal plastid genomes, thus demonstrating successive EGT from red algal plastid to red algal nucleus (nucleomorph) to stramenopile host nucleus [42]. Moreover, Moustafa and collaborators [35] provided evidence for a green algal-like endosymbiont in the common ancestor of chromalveolates as supported by the fact the 70% of diatom genes of Plantae origin are of green algae lineage provenance and that such genes are also found in the genome of other stramenopiles. Therefore, the authors postulate that chromalveolates were “green” prior to becoming “red” and are the result of serial secondary endosymbioses that conserved the genomic footprints of the previous endosymbioses, but finally displaced the plastid of green algal origin. However, the dataset used for comparison was biased against sequences of red algal origin. Indeed, the only complete or nearly complete red algal genomes available to date come from the acidothermophiles *Cyanidioshyzon merolae* [58] and *Galdieria sulphuraria *[59] which contain reduced gene sets (only 5,331 genes in *C. merolae*). Therefore, such analysis is likely to gain refinement by taking into account the forthcoming sequences of larger red algal genomes such as those from *Chondrus crispus* and *Porphyra umbilicalis* that are currently being assembled at Genoscope, France, and at the Joint Genome Institute (JGI), USA, respectively (Boyen C., personal communication; [60]).

Independently of an endosymbiotic event, horizontal gene transfer (HGT) between organisms has a major role in species evolution. Although commonly observed among bacteria, archaea, and between bacteria and archeae, it is much less frequent in eukaryotes [61,62]. Unexpectedly, analysis of the *P. tricornutum *proteome revealed that as much as 5% of its predicted genes were most closely related to bacterial genes, greatly surpassing the level of HGT previously described in eukaryotes. About half of those genes of putative bacterial origin were also found in the *T. pseudonana *genome, attesting their ancient incorporation in the diatom lineage [43]. Hence, stramenopile proteomes appear to consist of a melting pot of genes of various origins.

The *E. siliculosus* genome sequence includes 16,256 protein-coding genes that on average contain 7 introns per gene. Genome analysis has revealed the presence of an extensive set of light-harvesting and pigment biosynthesis genes that may explain brown algal fitness in tidal environments. Comparative genomics also revealed the emergence of a rich assortment of signal transduction genes including a group of membrane-spanning receptor kinases in brown algae since divergence from diatoms [45]. Interestingly, such receptor kinases play key roles in developmental processes including cellular patterning [63] in the multicellular animal and green plant lineages in which they evolved independently [64, 65]. The evolution of membrane-spanning receptor kinase in at least three of the five groups that have evolved complex multicellularity may therefore reflect a convergent evolution towards developmental sophistication [45].

Regulation of gene expression also seems to have evolved independently in each stramenopile lineage. A recent comparative study of transcription factors (TFs) in sequenced stramenopile genomes found that the Heat Shock Factor (HSF) family of TFs has massively expanded in the diatom lineage, where HSF genes account for about 35% of the TFs in both centric and pennate diatoms. The second and third largest families in diatoms are Myb and C2H2-Zn finger families of TFs, which are the most abundant TF classes in *E. siliculosus* and other stramenopiles [66].

## THE MAS JUNK

Transposable elements (TEs) are mobile genetic sequences that represent a variable fraction of most eukaryotic genomes such as 3% in baker’s yeast, 45% in our own, and over 80% in maize. TEs are thought to be important contributors to genome evolution by inserting in genes or genetic regulatory elements, thereby disrupting gene function or altering expression levels, triggering chromosomal rearrangements, and influencing the physical genome size. In order to moderate the impact of TEs on populations, genomes have evolved specific epigenetic defense strategies that limit their activity. Indeed, in most eukaryotic genomes, TEs and other repeat-like sequences show characteristic epigenetic features that commonly involve RNAi-mediated DNA methylation and specific histone modifications such as di-methylation of H3(K9) ([67]; Rivarola *et al*., unpublished results). Therefore, the identification and mapping of repeat sequences is a crucial step towards understanding the epigenetic landscape of a genome.

There are two main approaches to identify TEs in a genome: sequence similarity-based methods that scan genomic sequences for known elements or TE-characteristic structural features, and *de novo* methods that identify overrepresented sequences in a genome.

Several sequence similarity-based approaches have recently been applied to diatom genomes with the aid of programs that detect sequence repeats (i.e. RepeatMasker program [A.F.A. Smit, R. Hubley & P. Green RepeatMasker at http://repeatmasker.org]) in order to map the matching sequences on diatom genomes and to assess the abundance of each class of TE [43, 68]. It appears from these analyses that the TEs identified cover 6.4 and 1.9% of the *P. tricornutum* and *T. pseudonana *genomes, respectively, being long terminal repeat-retrotransposons (LTR-RT) the most abundant ones, accounting for 90% and 58% of the respective TE sequences. Diatom-specific *Copia*-type LTR-retrotransposons (LTR-RT) called CoDis have been significantly amplified in *P. tricornutum* with respect to *T. pseudonana*, constituting 5.8 and 1% of the respective genomes [43, 68]. Some copies of *Gypsy*-like LTR-RT were also found in *T. pseudonana* but not in *P. tricornutum*. However, *Gypsy*-like LTR-RT are not thought to be absent from all pennate genomes as corresponding sequences were found in cDNA libraries prepared from the pennate species *Pseudonitzschia multistriata* and *Pseudonitzschia multiseries*. Two thirds of the remaining TEs from *T. pseudonana* are composed by *Harbinger*-related DNA transposons that were not found in *P. tricornutum*.

The potential impact of CoDis on genome dynamics has been studied in more depth. Interestingly, transcription of two CoDi elements from *P. tricornutum* called *Blackbeard* and *Surcouf* was activated in response to nitrate starvation and to treatment with diatom-produced aldehydes, respectively, which constitute two ecologically relevant conditions [68]. The recent activity of CoDis in *P. tricornutum* species was demonstrated by revealing insertion polymorphism across several ecotypes. Furthermore, genomic features suggested the presence of TE-mediated recombination events that may have played a role in the evolution of gene families [66, 68]. Few additional repeats including potential TEs were identified using *de novo* approaches, suggesting that yet unclassified repeats constitute a limited fraction of diatom repeat content (Maumus *et al*., unpublished). Repetitive element analysis in larger diatom genomes may reveal the presence of other types of TEs that were present in a common ancestor and may have been lost in *P. tricornutum* and *T. pseudonana* as a consequence of genome reduction.

In the case of the* E. siliculosus* genome, a pipeline using a combination of multiple *de novo* and similarity-based programs [69, 70] and aiming at identifying and annotating repeated sequences in whole genome sequences was applied [45]. Manual curation confirmed the presence of different types of LTR-RT including *Ty1/copia*, *Ty3/gypsy*, and DIRS*/Ngaro*-like elements, as well as non-LTR retrotransposons (LINEs), and non-autonomous retrotransposable elements (TRIMs/LARDs). In addition, the presence of a novel type of LTR-RT dubbed large GAG-related elements (LGA) was reported [45]. LGAs present typical LTR-RT structural features and a ~400 aa open reading frame with a predicted C-terminal zinc finger that shares weak similarity with those from retroviral proteins. It remains to be determined if these proteins enable LGA elements to transpose autonomously. DNA transposable elements were also found including *Harbinger*, JERKY, POGO-like, and *Helitrons*. Altogether, the classified TE sequences were found to cover ~12.5 % of the *E. siliculosus* genome comprising ~9.4% from retrotransposable elements. In addition many unclassified repeats were identified and found to compose ~9.9% of the genome. Interestingly, one of those, denoted as “*Sower*”, was found to be the most abundant repeated sequence covering as much as 1.2% of the genome (~2.5 Mb) [45].

## DNA METHYLATION IN MAS

**Fig. (3):**
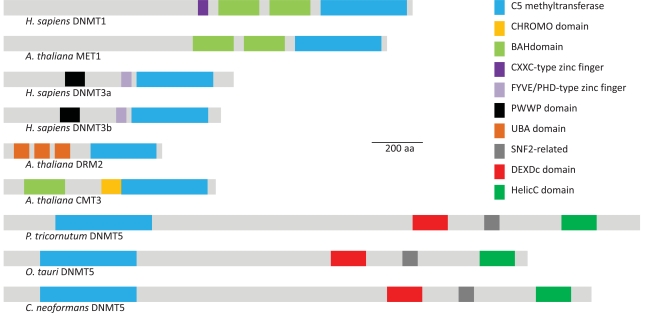
**DNMTs domains.** Schematic representation of the conserved domains found in various types of DNMTs including the DNMT5 family.

DNA (cytosine-5) methyltransferases (C5-MTases) are the enzymes that catalyze the transfer of a methyl group from S-adenosyl-l-methionine to the C5 position of cytosine residues in DNA. Functionally, C5-MTases are classified following two types of activities, namely “maintenance” and “*de novo*” methylation [71, 72]. Maintenance methylation occurs after DNA replication on hemimethylated symmetric motifs (CpG and CpNpG), whereas *de novo* methylation occurs at previously unmethylated cytosines (in symmetrical or asymmetrical sites). Eukaryotic C5-MTases can be grouped into several classes based on sequence similarity within their C-terminal catalytic domains and are mostly represented by the mammalian DNMT1, DNMT2, and DNMT3a/3b, and the chromomethylases (CMTs) which are specific to flowering plants [73]. In addition to the catalytic domain that contains the highly conserved C5-MTase motifs, these enzymes can contain extra domains which are believed to define the structural basis for the differences in biological functions (Fig. **3**). On the other side, DNMT2 homologs, which are present in most eukaryotes, were shown to methylate tRNA instead of DNA [74]. In addition, other types of C5-MTases such as DNMT4, DNMT5, and DNMT6 have been described in specific eukaryotic groups [75].

**Fig. (4):**
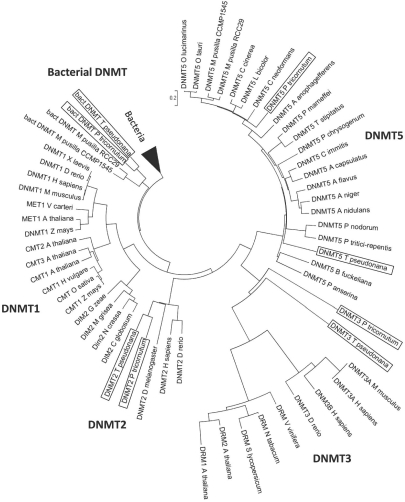
**DNMTs phylogenetic reconstruction.** Bootstrap-supported phylogenetic tree constructed with the Neighbor-Joining method (1000 iterations) showing the relationships between the DNA methyltransferase domains of selected DNMT families. Bacterial DNMTs, indicated by a black dashed curve, come from the following species: *Bacillus subtilis*, *Clostridium cellulolyticum* H10, *Clostridium kluyveri* DSM 555, *Moraxella sp*., *Legionella pneumophila* str. Paris, *Legionella pneumophila* str. *Lens, Shewanella* sp. MR-4, *Roseobacter litoralis* Och 149, *Neisseria meningitidis* MC58, *Sulfurimonas denitrificans* DSM 1251, *Bacteroides fragilis* YCH46, *Microcystis aeruginosa* PCC 7806, *Microcystis aeruginosa* NIES-843, *Dactylococcopsis salina*, *Cyanothece sp*. ATCC 51142, *Collinsella stercoris* DSM 13279, and *Mycoplasma arthritidis* 158L3-1. Fungal DNMT5 proteins, indicated by a grey dashed curve, come from the following species: *Ajellomyces capsulatus* NAm1, *Aspergillus flavus* NRRL3357, *Aspergillus nidulans* FGSC A4, *Aspergillus niger*, *Botryotinia fuckeliana* B05.10, *Coccidioides immitis* RS, *Coprinopsis cinerea* okayama7 (#130), *Cryptococcus neoformans var. neoformans* B3501A, *Laccaria bicolor* S238NH82, *Penicillium chrysogenum* Wisconsin 541255, *Penicillium marneffei* ATCC 18224, *Phaeosphaeria nodorum* SN15, *Podospora anserina*, *Pyrenophora triticirepentis* Pt1CBFP, and *Talaromyces stipitatus* ATCC 10500. The tree was drawn using the SplitsTree4 software [107].

Both the *T. pseudonana* and the *P. tricornutum* genomes contain four gene models with C5-MTase domain (Maumus *et al*., unpublished). Predicted proteins showing sequence similarity to members of the DNMT2 (Thaps3|22139 and Phatr2|16674) and DNMT3 (Thaps3|9575 and Phatr2|46156) gene families were detected in each genome (Fig. **4**). Also, as in most eukaryotes apart from plants and metazoans [75], DNMT1 homologs were not found in diatom genomes. Interestingly, both diatom genomes contain predicted proteins similar to members of the DNMT5 family (Thaps3|3158 and Phatr2|45071-45072), commonly found in ascomycete and basidiomycete fungi [75], as well as in the Pelagophyceae *Aureococcus anophagefferens* and the Prasinophyceae (green algae)* Ostreococcus tauri*, *Ostreococcus lucimarinus*, and *Micromonas pusilla* (Maumus *et al*., unpublished) (Fig. **4**). In contrast to other classes of eukaryotic DNMTs that present an extension in their N-terminal region, DNMT5 proteins contain a SNF2-type DEXDc/HELICc helicase domain at the C-terminus (Fig. **3**). Phylogenetic reconstruction and taxonomic distribution suggest that extant DNMT5-type proteins were present in a common eukaryotic ancestor. Interestingly, members of the SWI2/SNF2 family of ATP-dependent chromatin remodeling factors are necessary for proper cytosine methylation in plants and animals [76, 77] and they interact with DNMTs to repress transcription [78]. The direct involvement of certain SWI2/SNF2 helicases in DNA methylation processes and the conservation of their domain composition across eukaryotic groups suggest that DNMT5-like C5-MTases play a conserved and specific role in DNA methylation.

Phylogenetic analysis revealed that, in both genomes, a fourth predicted gene with C5-MTase domain appears to have evolved more recently from bacterial C5-MTases. Interestingly, these proteins are similar to sequences from *Micromonas* species (Fig. **4**) (Maumus *et al*., unpublished). Such evolutionary relationships suggest that these proteins have been transferred from bacteria to eukaryotes and then between eukaryotes. Their presence in both diatoms suggests a common ancestry and their absence in the remainder of the green lineage argues in favor of a transfer from extant diatoms towards extant prasinophytes.

The occurrence of DNA methylation in diatoms was first assessed by Jarvis and collaborators who used HPLC to analyze DNA nucleoside composition in several species of microalgae [79]. The authors described low levels of methylation in the DNA from two *P. tricornutum* ecotypes studied for which they reported a molar percentage for C5-methylcytosine of 0.11 and 0.16%, respectively. In the same study, DNA from other diatoms was shown to contain a higher 5mC molar percentage, up to 2.23% for the centric *Cyclotella cryptica* [79]. In contrast, preliminary genome-wide DNA methylation analysis of *P. tricornutum* using BS-seq showed that ~2.5% of all CpG motifs were methylated (Rivarola *et al*., unpublished). It will be interesting to re-investigate global DNA methylation levels and patterns in the genomes of more microalgae using the BS-seq method. Indeed, although repetitive DNA is ubiquitously methylated in most eukaryotes, 5mC has also been observed in protein-coding genes in the DNA of various eukaryotic groups [25, 26], although its function and regulation remains insufficiently understood. In this line, preliminary analysis of results from an experiment aiming at unveiling genome-wide DNA methylation pattern in the *P. tricornutum* genome followed by experimental validation suggests that a few genes are densely methylated in the genome (Maumus *et al*. and Rivarola *et al*., unpublished). Furthermore, recent use of the Mcr-PCR method to address the levels of DNA methylation in the *P. tricornutum* genome at specific loci also showed that most TEs were highly methylated in the *P. tricornutum* genome [68]. Unexpectedly, hypomethylation of the retrotransposon *Blackbeard* was detected and accompanied by its transcriptional activation in response to nitrate starvation [68]. It remains to be determined whether this observation was due to a failure to maintain DNA methylation after subsequent cell divisions or to an active demethylation mechanism acting at this locus.

In strike contrast, no putative DNA C5-MTase could be detected in the *E. siliculosus* genome where only a DNMT2 homolog was found therefore suggesting that this organism may lack cytosine methylation in nuclear DNA completely. Indeed, HPLC analysis as well as Mcr-PCR experiments targeting TE loci failed to detect 5mC in this genome [45]. Therefore, TE silencing mechanisms in *E. siliculosus* are likely to be primarily sustained by other processes including histone modifications. It will be interesting to decipher whether the lack of 5mC is a brown algal-shared feature or if Ectocarpales abandoned this process later in evolution and what alternative strategies were evolved.

## HISTONE MODIFICATIONS IN MAS

There are over 60 different residues on histones where modifications have been detected either by specific antibodies or by mass spectrometry [17]. The characterization of the enzymes catalyzing histone modifications has been the focus of intense research over the last 10 years. The enzymes that are responsible for eight different types of histone modifications including acetylation, methylation, phosphorylation, and ubiquitylation have been identified.

In order to provide a view of the possible histone modifications occurring in MAS genomes, we have searched for homologs of histone modifiers among the respective sets of predicted proteins. A rich repertoire of proteins with significant similarities to known histone modifiers was found in the three sequenced MAS genomes (Table **2**), such as putative homologs of the lysine acyltransferases (KAT) KAT1 and KAT2 [80, 81], CBP/p300 [82], MYST [83], and ELP3 (elongator complex protein 3) [84]. Putative class I, II, and III histone deacetylases (HDACs) such as RPD3 were also found in these genomes. Thus, dynamic acetylation/deacetylation of histones is likely to occur in these species. Preliminary data from ChIP-seq experiments on *P. tricornutum* utilizing specific antibodies to different modified histones under low and normal nitrogen conditions showed the expected dynamic nature of histone modifications (Rivarola *et al*., unpublished results).

Various putative lysine methyltransferases were also identified in these species including candidates for ASH1 (absent, small, or homeotic 1), and SETD2 homologs, as well as MLL-like and SMYD-like proteins which appear to be more abundant in *P. tricornutum* and *E. siliculosus*, respectively. Other types of putative histone lysine methyltransferases were found to be species-specific. For example, putative homologs of SETD1, SETDB1, EHMT2 (Euchromatic Histone-lysine N-MethylTransferase 2) [85], and SETMAR proteins were only found in the *E. siliculosus* genome. In addition, the brown algal genome also encodes two Trithorax-like proteins as well as a group of three TRX-related proteins, whose C-terminus displays high similarity to the SET domain of the TRX candidates. In *P. tricornutum*, we also found a gene model encoding a protein containing a SET domain and a Jumonji C (JmjC) domain. The existence of transcripts encoding proteins containing both domains was confirmed by PCR on *P. tricornutum* cDNA (data not shown). The presence of such a multi-domain protein is of particular interest because it links two antagonistic activities: SET which is known to mediate lysine methylation, and JmjC that mediates lysine demethylation. This putative protein may have a dual activity, perhaps on different residues of the same histone. Intriguingly, such a SET-JmjC fusion is also found within the fungus *Neurospora crassa* but in the opposite configuration (JmjC-SET).

In the two diatoms, the histone lysine demethylase JmjC domain was also found in two combinations with other domains. One putative protein contains JmjC and MBT (Malignant Brain Tumor) domains. Interestingly, the MBT domain has been reported to bind mono-methylated H3 (K4) and different degrees of methylation on H4 (K20) depending on the MBT domain tested [86]. Furthermore, it was recently shown that the *Drosophila* L3MBTL1 protein with MBT domain binds at least two nucleosomes, thus compacting nucleosomal arrays depending upon mono- and dimethylation of H4 (K20) [87]. Another domain found with JmjC in the same putative protein was the CHROMO domain. Along this line, CHROMO domain has been shown to interact with mono-, di-, and trimethylated H3 (K9) [88]. Furthermore, it was recently shown in fission yeast that the CHROMO domain-containing protein Swi6/HP1 interacts with the JmjC-domain-only protein Epe1 [89]. The function of MBT and CHROMO domains may be to address the histone demethylase JmjC activity to nucleosomes harboring specific histone marks. In addition, homologs of the JmjC-JmjN-containing protein JMJD2 were found in the three MAS genomes. Two other putative histone demethylase, LSD1 and FBXL homologs, were detected in the *P. tricornutum* and the *E. siliculosus* genomes. The presence of such a variety of histone methylases and demethylases in MAS genomes reinforces the dynamic histone methylation/demethylation that occurs on multiple lysine residues, as evident from our preliminary data from ChIP-seq experiments. In addition, we found in the three MAS genomes, genes encoding putative arginine methyltransferases, including various classes of PRMTs (protein arginine methyltransferase) that may act on different H3 and H4 arginine residues. Again, we also identified proteins with homology to the histone arginine demethylase JMJD6 [90] in all three genomes. The in vivo occurrence of modified histones was verified by Western blot and as mentioned above, by Chip-seq experiments using *P. tricornutum.* (Maumus *et al*. and Rivarola *et al*., unpublished results).

## RNA INTERFERENCE IN MAS

Small (20-30 nt) RNAs are fundamental, sequence-specific regulatory elements in eukaryotes. They are processed from double-stranded RNA (dsRNA) with perfect or near-perfect complementarity. dsRNA can have a multitude of origins that can vary across organisms. For example in plants, dsRNA can come from the secondary structure of mRNA containing inverted repeats generating a hairpin or a stem-loop (miRNA) [91, 20], the annealing of complementary transcripts (siRNA) [92], or RNA-directed RNA polymerization (RdRP) using as template AGO-sliced mRNA including trans-acting siRNA transcripts [93]. Other sources of dsRNA are protein-coding transcripts (secondary siRNAs) [94], or RdRP-synthesized RNAs using RNA polymerase IV transcripts as templates (repeat-associated siRNA) [95]. Such dsRNA can be cleaved into 20-30 nt dsRNA by RNase III-like enzymes (typically Dicer-like (DCL) or Drosha-like) that can vary according to the type of dsRNA substrate. One strand (called the guide strand) is then loaded onto an Argonaute-like (AGO) protein that acts in an RNA-induced silencing complex (RISC) to ensure sequence-specific mRNA cleavage or translational inhibition, or in other complexes that act in the nucleus to direct sequence-specific cytosine methylation and histone modifications. In addition to the AGO/RNase III-associated pathways, another small RNA silencing system, called Piwi-associated RNAs (piRNAs), has been shown to prevent the amplification of selfish genetic elements in animals [94].

Although the transformation of diatom nuclear genomes is now routinely achieved [52], the lack of tools to generate specific knock-out or knock-down mutants has remained a major limitation for functional genomics studies in diatoms. The RNA interference pathway is often exploited to down-regulate target genes [96]. However, because published diatom genomes do not encode proteins with extensive similarities to Dicer-like proteins [97], it remained obscure whether such a machinery exists in diatoms until it was proven experimentally [54]. In this work, a *P. tricornutum* transgenic line that expressed the GUS reporter gene under a strong promoter was transformed with anti-sense or inverted repeat sequences of the GUS gene and resulted in a significant down-regulation of GUS in several clones for both types of constructs, thus demonstrating the existence of an RNAi-based silencing system in *P. tricornutum*. It is however unclear whether down-regulation of the GUS gene occurs through transcript cleavage, translational inhibition or RdDM processes. Interestingly, Mcr-PCR analysis of the resulting down-regulated lines revealed the presence of DNA methylation in the GUS gene. Furthermore, bisulfite-sequencing uncovered a high density of 5mC in the targeted region of the GUS gene as well as a discrete 5mc distribution up to the promoter [54]. This suggests that in this case, siRNA-mediated down-regulation of the GUS gene involves at least an RdDM process and that *de novo* DNA methylation is able to spread from 5mC-rich loci in *P. tricornutum* following a yet to be determined mechanism. Also, it remains to be clarified whether other siRNA-mediated processes are involved in the down-regulation of the GUS gene. Interestingly, this technique has also been proved to be efficient to down-regulate the expression of endogenous genes [54, 98] and thus constitutes a robust tool for reverse genetics in diatoms. Additional experiments will also be required to explore the existence of other silencing mechanisms in diatoms.

On the basis of sequence similarity with known core proteins of the eukaryotic RNAi machinery, we identified several predicted proteins in diatom genomes that may be involved in RNAi-based mechanisms. First, similar to what was observed by Cerruti & Casas-Mollano [97] for *T. pseudonana*, homologs of the canonical Dicer proteins could not be found in either of the published diatom genomes. However, although canonical DCLs are large proteins that typically contain an N-terminal DEADc/HELICASEc domain followed by a domain of unknown function (DUF283), a PAZ domain, two neighboring RNase III domains (RNase IIIa and RNase IIIb), and a double-stranded RNA-binding domain (dsRBD), in some eukaryotes and especially protists, less complex RNase IIIa-IIIb- containing proteins were found to be capable of slicer activity. For instance, DCL1 from the ciliate *Tetrahymena thermophila *(alveolate) contains only the two RNase III domains associated to a dsRBD domain [99]. In *Giardia intestinalis *(excavate) DCL1, the two RNase III domains associate with a PAZ and a DUF283 domain [100]. Also, DCL1 from the excavate* Trypanosoma brucei*, which consists of only two adjacent RNase III domains, is sufficient to drive the RNA interference pathway [101]. In fact, although they have evolved from a different branch of ancestral RNase III, RNase III-like proteins (RTLs) are also found in the genomes of “higher” eukaryotes including plants where they may also play a role in small RNA biogenesis in addition to the typical DCLs [102]. Such RTL proteins with putative *dicing* activity were identified in *P. tricornutum* and *T. pseudonana*: in the former, a gene model encodes a protein with dsRBD followed by two RNase III domains, and in the latter, a predicted protein with only two RNase III domains. For both proteins, the conservation of key amino acid residues supports their role as bona fide dsRNA dicers. Interestingly, *T.pseudonana *also encodes a protein with a DEADc/HELICASEc domain and a C-terminal dsRBD [54]. A protein with such domain composition could not be identified in other organisms and it is tempting to speculate a role in the RNAi machinery. In addition, one AGO-like protein with canonical domain composition has been identified in both published diatom genomes but Piwi-like proteins could not be found. Nevertheless, proteins with similarity to RdRPs were found: one in *P. tricornutum* and two in *T. pseudonana* [54]. However, although quite a similar set of putative core RNAi proteins were found in both the pennate and centric diatoms, the pennate diatom *Fragilariopsis cylindrus* appears to possess quite a diversified complement of such proteins (Mock *et* *al*., unpublished).

In contrast to diatoms, the *E. siliculosus* genome was found to encode a canonical Dicer-like protein as well as one AGO and two RdRP homologs [45]. The *E. siliculosus* genome project also performed deep sequencing of small RNAs in this species. When small RNA sequences were mapped on the genome it was found that they were distributed primarily on rRNA sequences (45%), followed by intergenic regions (26%), TEs and other repeated sequences (13%) and introns (9%). Size analysis revealed enrichment in 21 nt species for the small RNAs that map on intergenic regions, repeated sequences, introns and exons [45]. Interestingly, there was a statistically significant link between small RNAs and repeated sequences suggesting the existence of chromatin-level small RNA-directed histone modifications in this species that controls TE amplification.* E. siliculosus* small RNA data were successfully combined with secondary structure prediction algorithms to screen the genome for putative miRNA genes where 26 miRNA sequences defining 21 families were identified. Most of the mature miRNA sequences are 20 nt long and begin with a U which is a well known characteristic of miRNA loaded on the AGO1 protein in plants. Target prediction revealed 71 potential transcript targets for 12 out of the 26 miRNAs. Interestingly, most (75%) of the predicted targets contain leucine-rich repeat domains [45].

## DISCUSSION AND FUTURE PERSPECTIVES

Diatom genomes are well on their way. These organisms, long studied by marine biologists for their morphology, phylogeny, and ecological success, are now providing valuable information into epigenetic processes such as transposon behavior in genomes, and the evolutionary history of eukaryotes. The recent sequencing of the three MAS species (plus a few more soon to be finished) 'have' and 'are' providing a valuable resource from which to study different biological phenomena previously unreachable, opening up a whole new arena of possibilities. Scientists can now tackle fundamental questions of biology in a genome-wide manner with many 're-sequencing' techniques, that use the reference genome to aid in mapping of the reads. The ability to perform ChIP-seq assays with commercial antibodies that detect a vast array of different histone modifications and BS-seq experiments provide a clear quantitative landscape of the particular chromatin modification in study. On the other hand, if this is coupled with RNA-seq, which provides an approximate snapshot of gene expression levels, a better understanding of how epigenetics can alter gene expression can be obtained.

To date, several diatom genomes are close to being completed: *Pseudo-nitzschia multiseries* (~250 Mb genome size) and *Fragilariopsis cylindrus* (~81 Mb genome size) are being sequenced by the Joint Genome Institute (USA: http://www.jgi.doe.gov/genome-projects), and *Amphora sp. *CCMP2378 and *Atheya sp.* CCMP212 are being sequenced by J. Raymond, U. Nevada, USA (personal communication). Also, the genome of the pelagophyte* Aureococcus anophagefferens* sequenced at JGI has been published [103] meanwhile the resubmission of this manuscript and provides the first complete genomic data from the MAS clade *Pelagophyceae*.

This genomic revolution has provided solid ground to ask some exciting questions, some of which are being addressed in our labs. One such example is determining the gene expression and epigenetic landscapes in different MAS species and the effect of environmental changes at a genome-wide level. Moreover, one can study the epigenetic modification stability after an environmental cue by examining the cells once they return to their normal condition (e.g. nitrogen-deficient medium, which is known to alter DNA methylation and transcription of certain transposons). Besides nutrient conditions, a very exciting area is to analyze the potential epigenetic remodeling that accompanies morphotype transition of *P. tricornutum* cells such as the switch in morphology from ‘fusiform’ to ‘round’ cell shape during biofilm formation [104]*.* The round cell morphotype is of particular interest because it may represent a cyst-like resting stage. Comparing the epigenomes of the three different *P. tricornutum* morphotypes may pinpoint different epigenetic profiles at specific genes that cause morphotype-specific expression levels and networks involved the regulation of morphological and metabolic processes [105]. These experiments will provide insight into how epigenetic changes influence gene expression and how environmental cues act upon chromatin. Moreover, the sequencing of *Pseudo-nitzschia multiseries, *another pennate diatom whose genome is 250 Mb (aprox. 10 times larger that *P. tricornutum*) will provide a great system to compare the epigenomes of these two diatoms.

Another interesting area of biology that can be assessed using MAS species is DNA replication. How origins of DNA replication in eukaryotes are specified is a fundamental biological question that remains unresolved. No DNA consensus sequence that allows initiation of DNA replication has been identified in eukaryotes, with the exception of *Saccharomyces cerevisiae*. Therefore, epigenetic and/or functional elements may be responsible for origin specification. Several studies have correlated origins of replication with transcription or epigenetic marks. Interestingly, transposable elements have also been associated with the presence or timing of origins of replication. The unique characteristics of MAS species, with contrasting DNA methylation and histone modification profiles, represent unicellular experimental systems that can be synchronized to study DNA replication origins. The nature of centromeres and kinetochores also represents an exciting topic because of the unusual nature of mitosis in MAS organisms such as diatoms [106].

Moreover, with the upcoming 3rd generation of DNA sequencers such as from PacBio (http://www.pacificbiosciences.com), that will provide up to several Kb read lengths, assembling new diatom genomes will be faster, more accurate, and hopefully less expensive. A safe assumption is that in the near future tons of data from marine organisms will be produced and the challenge to analyze all that information will be daunting. The unique characteristics of the MAS species and all the 'omics' data that will come make it a great time to be in the marine world.

## Figures and Tables

**Table 1:** Major Features of the *P. tricornutum*, *T. pseudonana* and *E. siliculosus* Genomes

	*P. tricornutum*	*T. pseudonana*	*E. siliculosus*
Sequencing center	JGI	JGI	VIB/Ugent
Genome size	27.4 Mb	32.4 Mb	195.8 Mb
Predicted genes	10,402	11,776	16,256
Introns per gene	0.79	1.52	6.98
Bacterial genes*	587	> 328	NA
Known TEs	6.4%	1.9%	12.5%
Other repeats*	NA	NA	9,9%

* For *T. pseudonana*, the number indicates that although species-specific estimation is not available, 56% of the *P. tircornutum* bacterial gene pool was also found in *T. pseudonana*. The number of bacterial genes was not yet estimated in *E. siliculosus*. The amount of unknown repeats in diatoms has not yet been estimated.

**Table 2:** Putative Histone Modifiers Found in MAS Genomes

Histone Modifiers	Residues Modified	Homologs in *P.tricornutum*	Homologs in *T. pseudonana*	Homologs in *E. siliculosus*
Lysine Acetyltransferases (KATs)				
HAT1 (KAT1)	H4 (K5, K12)	54343	1397, 22580	Esi0002_0151
GCN5 (KAT2)	H3 (K9, K14, K18, K23, K36)	46915	15161	Esi0090_0053
Nejire (KAT3); CBP/p300 (KAT3A/B)	H3 (K14, K18, K56) H4 (K5, K8); H2A (K5) H2B (K12, K15)	45703, 45764, 54505	24331, 269496, 263785	Esi0053_0101
MYST1 (KAT8)	H4 (K16)	24733, 24393	37928, 36275	Esi0090_0053, Esi0084_0032
ELP3 (KAT9)	H3	50848	9040	Esi0264_0014
**Lysine Deacetylases (HDACs)**				
RPD3 (Class I HDACS)	H2, H3, H4	51026, 49800	41025, 32098, 261393	Esi0147_0031, Esi0181_0051, Esi0092_0077
HDA1 (Class II HDACS)	H2, H3, H4	45906, 50482, 35869	268655, 269060, 3235, 15819	Esi0168_0016, Esi0157_0055, Esi0157_0057, Esi0040_0047
NAD+ dependant (Class III HDACS)	H4 (K16)	52135, 45850, 24866, 45909, 52718, 21543, 39523	269475, 264809, 16405, 35693, 264494, 16384, 35956	Esi0054_0036, Esi0014_0023, Esi0026_0040
**Lysine Methyltransferases**				
MLL	H3 (K4)	40183, 54436, 42693, 47328, 49473, 49476, 44935	35182, 35531, 22757	Esi0069_0089
ASH1/WHSC1	H3 (K4)	43275	264323	Esi0016_0129, Esi0000_0259
SETD1	H3 (K36), H4 (K20)	not found	not found	Esi0070_0045, Esi0020_0054, Esi0043_0074
SETD2	H3 (K36)	50375	35510	Esi0028_0153
SETDB1	H3 (K9)	not found	not found	Esi0162_0064
SETMAR	H3 (K4, K36)	not found	not found	Esi0100_0024
SMYD	H3 (K4)	bd1647, 43708	23831, 24988	Esi0013_0081, Esi0189_0013, Esi0189_0015, Esi0286_0007, Esi0015_0158
TRX-related		not found	not found	Esi0115_0076, Esi0076_0098, Esi0043_0074, Esi0094_0082, Esi0455_0010
E(Z)	H3 (K9, K27)	32817	268872	not found
EHMT2	H3 (K9, K27)	not found	not found	Esi0453_0004
SET+JmjC	Unknown	bd1647	not found	not found
**Lysine Demethylases (KDM)**				
LSD1 (KDM1)	H3 (K4, K9)	51708, 44106, 48603	not found	Esi0073_0073, Esi0060_0020, Esi0014_0196
FBXL (KDM2)	H3 (K36)	42595	not found	Esi0025_0159
JMJD2 (KDM4)/JARID	H3 (K9, K36)	48747	2137	Esi0145_0049, Esi0014_0020, Esi0084_0076
JMJ-MBT	Unknown	48109	22122	not found
JMJ-CHROMO	Unknown	Pt1-40322	1863	not found
**Arginine Methyltransferases**				
CARM1 (PRMT4)	H3 (R17)	17184	3690, 28185	Esi0137_0007, Esi0000_0236, Esi0165_0056
PRMT5	H3 (R8), H4 (R3)	49565	24429	Esi0006_0115
PRMT6	H3 (R2)	54710	bd1828	Esi0153_0004, Esi0000_0300
PRMT7	H4 (R3)	49245	25810	Esi0133_0084, Esi0122_0102
**Arginine demethylases**				
JMJD6	H3 (R2), H4 (R3)	3251, 35989, 46234	24024	Esi0055_0014, Esi0131_0061, Esi0189_0080, Esi0055_0061

Proteins with putative histone modifier function were identified in the sets of predicted proteins from the MAS genomes by reverse BLAST comparison with enzymes known to catalyze the modification of specific histone residues (as indicated) and by searching for corresponding catalytic InterPro domains in MAS annotation databases. Candidate proteins are indicated by their identification number.
